# Stress induced hypertensive response: should it be evaluated more carefully?

**DOI:** 10.1186/1476-7120-9-22

**Published:** 2011-08-16

**Authors:** Nagehan Kucukler, Fatih Yalçin, Theodore P Abraham, Mario J Garcia

**Affiliations:** 1 Johns Hopkins Medical Institutions, Department of Cardiology, Baltimore, MD, USA; 2 Montefiore Einstein Center for Heart and Vascular Care, Albert Einstein College of Medicine, New York, NY, USA

**Keywords:** exercise hypertension, exercise electrocardiography, myocardial perfusion imaging, dobutamine stress echocardiography

## Abstract

Various diagnostic methods have been used to evaluate hypertensive patients under physical and pharmacological stress. Several studies have shown that exercise hypertension has an independent, adverse impact on outcome; however, other prognostic studies have shown that exercise hypertension is a favorable prognostic indicator and associated with good outcome. Exercise hypertension may be encountered as a warning signal of hypertension at rest and future hypertensive left ventricular hypertrophy. The results of diagnostic stress tests support that hypertensive response to exercise is frequently associated with high rate-pressure product in hypertensives. In addition to the observations on high rate-pressure product and enhanced ventricular contractility in patients with hypertension, evaluation of myocardial contractility by Doppler tissue imaging has shown hyperdynamic myocardial function under pharmacological stress. These recent quantitative data in hypertensives suggest that hyperdynamic myocardial function and high rate-pressure product response to stress may be related to exaggerated hypertension, which may have more importance than that it has been already given in clinical practice.

## Introduction

Measurement of blood pressure at rest has been usually used in epidemiological studies [1]. Some other measurements have also been used in blood pressure evaluation including pressure readings in basal conditions [2], during ambulation [3] and during exercise [4]. The exercise hypertension is defined according to Framingham criteria, namely, a peak systolic blood pressure during exercise greater than 210 mm Hg in men and 190 mm Hg in women [5]. In healthy normotensive adults, increased blood pressure response to exercise may be associated with a higher risk of developing hypertension at rest [6] and increased incidence of hypertensive left ventricular hypertrophy [7,8].

### Prognostic importance

It is well documented in large prospective epidemiological trials that high blood pressure at rest results in fatal and nonfatal cardiovascular events [1]. There are conflicting data related with the prognostic importance of increased hypertensive response to exercise [8-10], some data showed that exercise hypertension has an independent, adverse impact on outcome [9,10]. However, similar findings have not always been achieved [8]. It has been suggested that blood pressure measured at maximal exercise is a better prognostic indicator than clinic pressure [4]. It has been also shown in a recent comprehensive study that exercise hypertension is associated with a lower mortality rate [11]. It has been shown in a large study that systolic pressure during exercise predicted total mortality independently of age; after additional adjustment for the pressure at sitting rest, only peak exercise pressure remained related to outcome; the adjusted diastolic exercise pressures did not predict mortality [12]. In a shorter follow-up, totaling 1573 patient years, the prognostic significance of exercise blood pressure were assessed and concluded that blood pressure at 50 W, at 50% of peak exercise, and at peak workload did not add prognostic precision to the pressure at rest [8]. But those findings have been disputed [13,14], but there are important differences between these studies and the previous report [8]: a large number of healthy middle-aged men versus a smaller number of referred hypertensive patients; noninvasive versus intra-arterial blood pressure measurements; a short versus a longer period of rest before exercise; a relatively steep exercise protocol versus progressive graded multistage exercise, as conventionally used for clinical purposes; and differences in the studied end points and statistical methods. Analysis, based on continued follow-up of those hypertensive patients, supports the earlier conclusion [8] that intra-arterial pressure at submaximal and peak bicycle exercise does not add prognostic precision to the pressure measured at rest before exercise, except for the small independent predictive value of peak systolic pressure for total mortality [12]. Fagard et al. have explained why exercise blood pressure seems to provide independent prognostic information in healthy middle-aged men [13,14] and not in selected hypertensive patients. It is conceivable that the positive association between outcome and an excessive blood pressure elevation during exercise observed in the population-based samples resulted from an attenuated exercise-induced vasodilatation, as suggested previously [14]. It can be argued that in contrast to hypertensive patients, healthy subjects have a normal cardiac output response to exercise. Consequently, an impaired reduction of systemic vascular resistance would not be opposed by a blunted rise of cardiac output and is therefore expressed in excessive blood pressure elevation. Regarding to that study; all-cause mortality was significantly related to intra-arterial pressure and systemic vascular resistance and not to cardiac output. The results are less consistent for the measurements during exercise, when only systemic vascular resistance at peak effort carried prognostic information over and above that of vascular resistance at rest. Moreover, the prognostic importance of vascular resistance was not opposed by cardiac output, so the prognostic precision of peak exercise pressure was independent of pressure at rest [12].

### Risk for future hypertension

Although some studies suggested that systolic blood pressure rises with dynamic exercise, while diastolic blood pressure does not change or falls slightly at submaximal exercise [15], in another study documented that an exaggerated diastolic blood pressure response to exercise was predictive of risk for new-onset hypertension in normotensive men and women [16]. Increased blood pressure response to exercise is remarkable in the patients with hypertension compared with normotensives [17]. It has been suggested that this type of response to exercise can be used as an additional risk marker for hypertension [6].

A finding of another study was a high prevalence of masked hypertension in apparently healthy patients presenting with normal office blood pressure but high blood pressure during a clinically indicated exercise stress test [18]. The prevalence of masked hypertension in hypertension response to exercise patients from that study was fourfold this value highlighting the importance of an exercise stress test as a screening tool for masked hypertension [18].

The findings that an individual's risk of developing hypertension in those with high-normal blood pressure was greatly increased if they exhibit an exaggerated blood pressure response to exercise confirms an incremental contribution of exercise blood pressure response above resting blood pressure in predicting future hypertension. Therefore, exercise testing in populations at high risk for hypertension could provide important additional information concerning hypertension risk [19].

### Myocardial Functions

A hypertensive response to exercise is associated with subtle systolic dysfunction, even in the absence of resting hypertension. These changes occur before the development of left ventricular hypertrophy or detectable diastolic dysfunction and likely represent early hypertensive heart disease [20].

An exaggerated increase in systolic blood pressure prolongs myocardial relaxation and increases left ventricular chamber stiffness, resulting in an increase in left ventricular filling pressure. Left ventricular filling pressures can be estimated reliably by combining mitral inflow early diastolic velocity (E) and annulus velocity (E'). An increased E/E' ratio reflects elevated filling pressures and may be useful in assessing an abnormal increase in filling pressures for patients with diastolic dysfunction. In a study to evaluate left ventricular diastolic dysfunction leading to exercise intolerance, even in the absence of resting hypertension, (129 subjects with a preserved ejection fraction and a negative stress test) hypertensive response to exercise was evaluated at the end of a 6-min exercise test using the modified Bruce protocol. It is shown that early diastolic mitral annular velocity (E') by tissue Doppler imaging was significantly lower and the ratio of early diastolic mitral inflow velocity (E) to E' (E/E') was significantly higher in patients of the hypertensive response to exercise. Irrespective of the presence of resting hypertension, patients with hypertensive response to exercise had impaired left ventricular longitudinal diastolic function and exercise intolerance [21]. Diastolic stress echocardiography using a supine bicycle is technically feasible for demonstrating changes in E/E' (filling pressure) with exercise. The results of a study have been suggested the hemodynamic consequences of exercise-induced increase in diastolic filling pressure can be demonstrated noninvasively with exercise Doppler echocardiography [22].

### Stress echocardiography

Various diagnostic methods have been used to evaluate hypertensive patients and there have been conflicting data relating the effectiveness of the diagnostic methods. Exercise electrocardiography has low specificity especially in patients who have electrocardiographic abnormalities at rest [23]. Dobutamine stress echocardiography has shown to have more specificity in patients with hypertension compared with myocardial perfusion imaging [24,25]. It has also shown that dobutamine stress echocardiography and single-photon emission computed tomography have similar accuracy in patients with hypertension [26].

Factors related to false-positive results of exercise echocardiography to diagnose coronary disease have been extensively studied and it has been suggested that the factors predictive of false-positive results during exercise echocardiography to diagnose coronary disease were female sex, higher blood pressure at peak exercise, lower risk by Duke score and lower number of abnormal segments and wall motion score index after peak exercise. Women appear to be susceptible to wall motion abnormalities caused by elevated exercise blood pressure [27].

**Figure 1:**
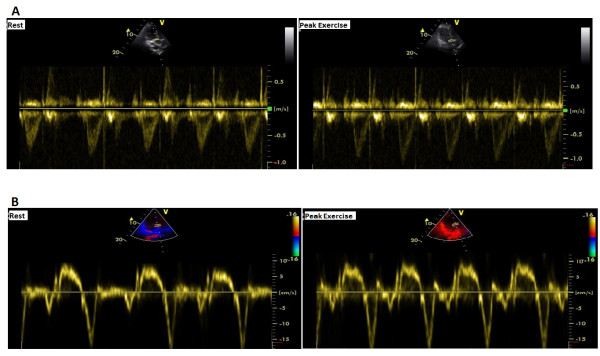
**Exercise stress echocardiographic images**. **A: **Measurements of left ventricular outflow tract velocities at rest and peak exercise, **B**: Basal septal tissue systolic velocities at rest and peak exercise, in a hypertensive 53-year-old woman with left ventricular hypertrophy [28].

We have recently documented increased regional myocardial contractility of left ventricular base using exercise stress echocardiography tissue Doppler imaging in hypertensives with left ventricular hypertrophy and relation between stress-related regional tissue dynamics and left ventricular outflow tract blood flow (Figure 1) [28]. Interestingly, it has been shown that the chronotropic response and the systolic blood pressure during Dobutamine stress echocardiography were higher in the patients with left ventricular obstruction. Dynamic left ventricular obstruction is common during Dobutamine stress echocardiography in a population of patients with angina-like chest pain without epicardial coronary artery disease and is associated with a higher hemodynamic responsiveness to dobutamine [29].

### Myocardial Contractility

**Figure 2:**
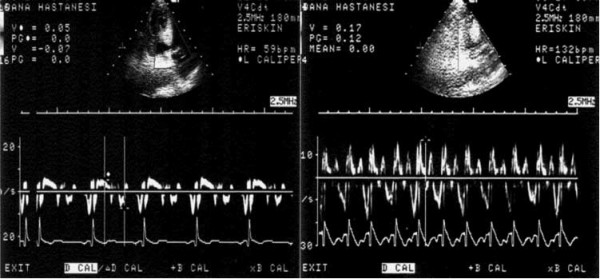
**Dobutamine stress echocardiographic images**. Measurements of maximum basal septal tissue systolic velocities at rest and peak stress in a hypertensive patient with basal septal hypertrophy [30].

We have observed that peak pharmacologic stress causes high systolic velocities on basal septal tissue in the hypertensive patients with basal septal hypertrophy suggesting stress may produce an enhanced left ventricular contractility (Figure 2) [30]. It has been suggested that enhanced left ventricular contractility exists in the patients with borderline blood pressure elevations [31]. In this study, the patients with higher ambulatory blood pressures had a greater velocity of fiber shortening in relation to end-systolic wall stress, indicative of an enhanced inotropic state. These findings may support some previous observations that excessive sympathetic stimulation [32] and left ventricular hypercontractile state [33] may be a part of the clinical spectrum of the hypertensive patients. Several previous studies demonstrating enhanced ventricular contractility in subjects with mild hypertension have analyzed ventricular function by relating endocardial fiber shortening to end-systolic stress [34].

Left ventricular end-systolic pressure-volume relationship provides a robust, relatively load-insensitive evaluation of contractility and can be assessed non-invasively during stress echocardiography. This index of global contractility is reasonably simple, does not affect the imaging time, and only minimally prolongs the off-line analysis time. It allows unmasking quite different, and heterogeneous, contractility reserve patterns underlying a given ejection fraction at rest [35]. Recently, a cutaneous force-frequency relation recording system has been validated based on heart sound amplitude and timing variations at increasing heart rates. Post-exercise contractility, diastolic time and pressure changes can be continuously measured by a cutaneous sensor. Monitored by a non-invasive wearable sensor, Bombardini et al. suggested that heart disease affects not only exercise systolic performance, but also post-exercise recovery, diastolic time intervals and blood pressure changes [36].

### Myocardial perfusion

Exaggerated hypertension is well tolerated and associated with only minor symptoms in most of the patients for the evaluation of hypertensives with chest pain [37]. In these patients, it has been suggested that inhomogeneous myocardial perfusion pattern in myocardial perfusion imaging may be caused by microvascular dysfunction [38].

Systemic hypertension and an exaggerated blood pressure response with exercise have been associated with 'false-positive' findings on stress electrocardiography and echocardiography [39]. In a cohort study of 7,205 patients who underwent stress testing with technetium 99 m-SPECT myocardial perfusion imaging (MPI) for the evaluation of chest pain or dyspnea, blood pressure responses to exercise were assessed. Then it has been shown that a hypertensive blood pressure response to exercise is not associated with increased rates of ischemic ECG changes, higher-risk Duke treadmill scores, greater degrees of abnormal MPI or worse clinical outcome. Those subjects with a hypertensive response to exercise have high, potentially excessive, rate-pressure products, which theoretically may result in global subendocardial ischemia due to myocardial oxygen demand exceeding supply [40].

### Nonischemic Cardiomyopathy

**Figure 3:**
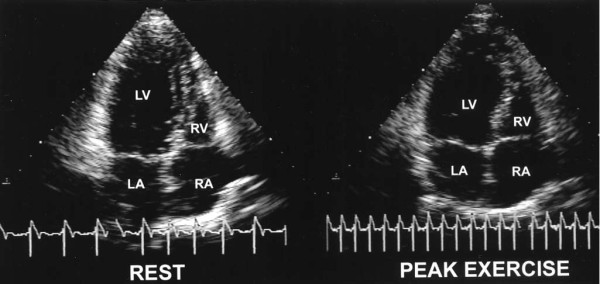
**Enlarged left ventricular cavity after peak exercise**. Exercise echocardiogram obtained from an apical four-chamber view of the heart in a 73-year-old woman with a hypertensive response to exercise. An end-systolic image taken immediately after peak exercise (right) shows an enlarged left ventricular (LV) cavity compared with image at rest (left). LA: left atrium; RA: right atrium; RV: right ventricle [39].

It has been reported that hypertensive patients who have well controlled blood pressure at rest may give exaggerated blood pressure response to exercise [8]. Hypertension is often cited as a cause of false-positive exercise electrocardiographic results [41-44]. Increased left ventricular afterload during an elevation in arterial systolic pressure may reduce the extent of ejection [39]. Therefore, the authors mentioned that exaggerated blood pressure response to exercise may result in greater likelihood of new or worsening abnormalities with exercise in the absence of angiographically significant coronary artery stenosis. In this study, it has been pointed out that potential sources of this condition may include normal heterogeneity in wall motion that is exaggerated during exercise or nonischemic cardiomyopathy. False-positive results were twice as likely in patients with a hypertensive response to exercise. The likelihood of false-positive results increased with increasing systolic and diastolic blood pressures. Recognition of this phenomenon is important for interpreting individual exercise echocardiograms (Figure 3) [39]. In another study of subjects referred for symptom-limited treadmill exercise testing and coronary angiography, hypertensive response to exercise predicted a lower prevalence of severe angiographic CAD and a lower adjusted mortality rate [45]. In clinical practice, findings of severe global hypokinesis after exercise may lead to a presumptive diagnosis of left main or severe multivessel CAD but may occur in association with normal findings on coronary angiography in some patients with a hypertensive response. Potential mechanisms by which a hypertensive response could cause abnormal wall motion may include an excessive rate-pressure product, which results in global subendocardial ischemia due to a mismatch between myocardial oxygen supply and demand. The potential for abnormal loading conditions to cause wall motion abnormalities is supported by the finding that healthy young adults can develop abnormal ventricular contractility by performing sudden vigorous exercise [46].

### End-organ involvement

It has been suggested that exaggerated blood pressure response during exercise testing may provide a foresight for the future of sustained hypertension development [47]. It is known that hypertension is associated with multiple end-organ involvement, including left ventricular hypertrophy; echocardiography provides a documentation of a relatively high frequency of left ventricular hypertrophy in even mild hypertension [48]. Even in the absence of hypertension, exaggerated blood pressure responses during exercise testing suggest a probability of left ventricular hypertrophy, a finding associated with the cardiac "end-organ" manifestations of hypertension [7,49]. In asymptomatic individuals, elevated exercise blood pressure carried higher risk of cardiovascular disease death but became nonsignificant after accounting for rest blood pressure [50].

It has been documented that the patients with exercise hypertension presented significantly greater posterior wall thickness and Doppler functional data showed significant increase in the late to early flow velocity ratio. Even in the absence of hypertension, exaggerated blood pressure reactivity to physical exercise suggests greater blood pressure elevation during daily activity as well as enhanced sympathetic nervous tonus, which may be considered a risk factor promoting hypertension and these hemodynamic and neurohumoral behaviors are associated with a tendency to develop target organ abnormalities in the heart [51].

### Mechanism responsible for the exercise hypertension

Although the mechanism responsible for the exaggerated blood pressure response to exercise has not been revealed, there are some plausible mechanisms linking with underlying structural abnormalities in the cardiovascular system. Wilson et al. [52] found that the total peripheral resistance in those with exercise hypertension did not fall adequately to compensate for the rise in cardiac output during exercise. Accordingly, the exercise hypertension can partially be explained by increased peripheral vascular resistance and impaired capacity for exercise induced vasodilatation. These responses of peripheral vascular function can be explained by a hyper-reactivity of sympathetic nerves and an increased vascular response to adrenergic stimulation or by a thickening of the arteriolar wall that alters its ability to respond to vasoconstrictor stimuli [53]. Among those patients with these characteristics, higher cardiac output not only raises the systolic blood pressure but also causes marked diastolic blood pressure elevations like those occurring in established hypertension. Moreover, several studies have found that normotensive individuals with hypertensive response to exercise present changes in the heart structure and function that are usually observed in the early course of the hypertension disease [7,54,55].

Lauer et al. [11] have detected that the patients with exercise hypertension have higher rate-systolic blood pressure product provocated by exercise. In this study, because the authors have detected that these patients had less severe angiographic coronary artery disease, they have speculated that the finding may represent an increase in myocardial oxygen demand. Higher rate-systolic blood pressure product may also be a reason for global subendocardial ischemia and subsequent false positive exercise test result due to a mismatch between myocardial oxygen supply and demand [39]. We have recently observed that pharmacologic stress results in exercise hypertension and higher rate-systolic blood pressure product in hypertensive patients with basal septal hypertrophy [56].

### Plausible treatments

Al-Nasser et al. [57] have shown that beta-blocker therapy have a beneficial effect on reduction of rate-systolic blood pressure product provocated by pharmacologic stress in patients with basal septal hypertrophy (all but one hypertensive). In a retrospective study the blood pressure response during exercise has been assessed in 2,318 hypertensive patients treated with various antihypertensive medications including angiotensin-converting enzyme inhibitors, calcium-channel blockers, diuretics, and combinations, beta-blockers alone or in combination with other antihypertensive agents. The findings support that the use of betablockers as monotherapy or in other antihypertensive agents mitigates the blood pressure response at maximal and absolute submaximal exercise workloads and could prevent an exaggerated blood pressure response in a considerable proportion of patients [58].

Some results indicate that an angiotensin converting enzyme inhibitor provides a sustained 24 h blood pressure reduction associated with blood pressure reduction during physical exercise. These effects have been obtained without altering diurnal blood pressure profile, without excessive hypotension at the time of maximal antihypertensive effect and with a slight improvement of exercise tolerance [59]. It has been found in patients with Doppler echocardiographic evidence of mild left ventricular diastolic dysfunction and a marked hypertensive response to exercise without resting hypertension that treatment with an angiotensin II receptor blocker blunted the increase in systolic blood pressure with exercise, improved exercise tolerance and enhanced the quality of life [60].

Franz et al. [61] examined an antihypertensive effect of endurance training on patients with essential hypertension and clearly showed not only a significant fall of resting blood pressure but also a marked reduction of the blood pressure and heart rate and, thus, of myocardial oxygen consumption during exercise after an endurance training program. Accordingly, it is suggested that endurance training would have the beneficial effect on modification of risk factors, especially when linking the exercise-induced hypertension [61].

## Conclusion

Recent quantitative data support that exercise hypertension in hypertensive patients may be related to enhanced ventricular contractility and excessive rate-pressure product response to stress and this finding may have more importance than that it has been already given in clinical practice.

## Competing interests

The authors declare that they have no competing interests.

## Authors' contributions

All authors contributed to the paper and meet the criteria for authorship. All authors read and approved the final manuscript.
